# Two-dimensional slither swimming of sperm within a micrometre of a surface

**DOI:** 10.1038/ncomms9703

**Published:** 2015-11-10

**Authors:** Reza Nosrati, Amine Driouchi, Christopher M. Yip, David Sinton

**Affiliations:** 1 Department of Mechanical and Industrial Engineering, University of Toronto, Toronto, Ontario, Canada M5S 3G8; 2 Department of Biochemistry, University of Toronto, Toronto, Ontario, Canada M5S 1A8; 3 Institute of Biomaterials and Biomedical Engineering, Terrence Donnelly Centre for Cellular and Biomolecular Research, University of Toronto, Toronto, Ontario, Canada M5S 3E1; 4 Department of Chemical Engineering and Applied Chemistry, University of Toronto, Toronto, Ontario, Canada M5S 3E5

## Abstract

Sperm motion near surfaces plays a crucial role in fertilization, but the nature of this motion has not been resolved. Using total internal reflection fluorescence microscopy, we selectively imaged motile human and bull sperm located within one micron of a surface, revealing a distinct two-dimensional (2D) ‘slither' swimming mode whereby the full cell length (50–80 μm) is confined within 1 μm of a surface. This behaviour is distinct from bulk and near-wall swimming modes where the flagellar wave is helical and the head continuously rotates. The slither mode is intermittent (∼1 s, ∼70 μm), and in human sperm, is observed only for viscosities over 20 mPa·s. Bull sperm are slower in this surface-confined swimming mode, owing to a decrease in their flagellar wave amplitude. In contrast, human sperm are ∼50% faster—suggesting a strategy that is well suited to the highly viscous and confined lumen within the human fallopian tube.

## Abstract (web summary)

Sperm motion near surfaces plays a key role in fertilization, but a description of how this motion differs from bulk swimming is lacking. Here, Nosrati *et al.* visualize sperm swimming within 1 μm of a glass surface and describe a ‘slither' swimming mode which differs from bulk helical swimming, and increases the velocity of human sperm.

## Graphical abstract

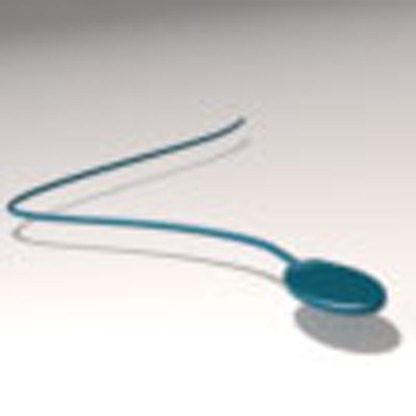

Sperm motion is central to natural reproduction. Sperm must traverse thousands of body lengths in the complex three-dimensional (3D) female reproductive tract to reach the egg^1^. During this journey, sperm exhibit a variety of motility modes (that is, motile, non-motile or hyperactivated) and swimming patterns (that is, typical, helical, hyper-helical, hyper-activated or chiral ribbons)—all of which are 3D in nature^2^,^3^. Such swimming patterns are the result of sperm flagellar motility generated by the dynein motor^4^. The dominant swimming pattern has been found to depend on geometrical, physiological, chemical and rheological stimuli present in the female reproductive tract^5^,^6^,^7^. The structure of the tract, such as the highly folded and ciliated epithelium in the oviduct^8^,^9^, presents a high-surface area, highly confined environment wherein surface effects have been shown to alter sperm motion^9^,^10^. The nature of sperm motion and the corresponding beating pattern within 1 μm of the surface are unknown.

The presence of solid boundaries results in the accumulation of sperm on surfaces^11^,^12^,^13^ due to a combination of hydrodynamic forces^14^,^15^ and steric repulsion^16^,^17^, a phenomenon known as surface accumulation behaviour. This phenomenon and its effects on microswimmer locomotion have been leveraged to select sperm with high DNA integrity^18^,^19^, and studied extensively through traditional microscopy^20^,^21^,^22^, mathematics^23^,^24^,^25^,^26^, computational fluid dynamics^27^,^28^,^29^ and biology^30^,^31^,^32^. Key aspects addressed by these studies are the extent to which surface accumulation is affected by: (i) geometrical or hydrodynamic constraints^12^,^33^, (ii) flagellar beat patterns and chiral components of the flagellar wave^31^,^32^, (iii) correlation between the near-wall circling direction of microswimmers and the rolling direction of the flagellar wave^32^,^34^ and (iv) microswimmer morphology^35^,^36^,^37^. Using dark-field microscopy, Woolley^32^ observed a planar waveform for ram sperm swimming near a glass surface in a medium with very high viscosity (1,500 mPa·s). This planar waveform near surfaces was used by Woolley *et al.*^38^ to study flagellar synchronization of bull sperm in a high-viscosity medium and also by Friedrich *et al.*^39^ to determine the drag characteristics of bull sperm using the resistive force theory. The inherent depth-of-field limitations of traditional optical microscopy, however, prevented observation of sperm–surface interactions at the scale of the individual cell.

Here, we report direct experimental observations of sperm swimming patterns within 1 μm of the wall. These experiments are enabled by imaging sperm using total internal reflection fluorescence (TIRF) microscopy. TIRF microscopy selectively illuminates the fluid–wall interface with the image intensity decaying exponentially with distance from the surface, enabling 3D tracking within a micron of the wall^40^,^41^. We find that sperm exhibit an intermittent two-dimensional (2D) swimming mode, whereby the full sperm length (50–80 μm) is confined within 1 μm from the surface. In this mode, the sperm head is aligned with the surface and tail beats are confined in the same 2D plane. These results suggest a distinct swimming mode for sperm, referred to here as the slither swimming mode, whereby sperm exhibit a 2D swimming pattern within 1 μm of a surface. This mode is in contrast to 3D rotational characteristics of established modes. We find that bull sperm exhibit this swimming mode even in low-viscosity media; however, for human sperm, slither swimming is prevalent only at higher viscosities (>20 mPa·s). While bull sperm are 50% slower in this surface-confined swimming mode, human sperm are 50% faster. Both bull and human sperm exhibit a straighter swimming trajectory in the slither mode. These findings indicate a distinct surface-confined sperm swimming mode that is well suited to the highly viscous and confined regions of the reproductive tract.

## Results

### The 2D slither swimming mode

**Figure 1:**
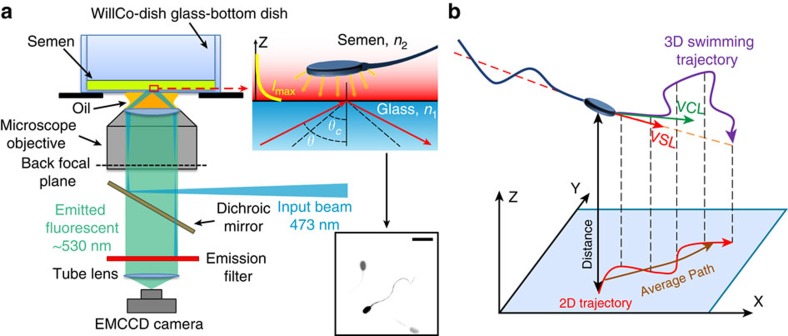
TIRF microscopy set-up for near-field imaging of sperm. (**a**) Schematic view of the TIRF microscopy set-up. A representative TIRF microscopy image showing both sperm head and tail for the sperm cell closest to the surface. Scale bar, 20 μm. The image intensity was inverted and contrast adjusted for clarity. (**b**) Cartesian coordinate system used to quantify sperm motion.

Both human and bull sperm were imaged in bulk liquid (away from the surface) and near the wall (within 1 μm from the surface) with epifluorescence and TIRF microscopy, respectively (Fig. 1a; Methods). Total internal reflection of the incident light at the glass–liquid interface results in a thin evanescent wave propagating in the liquid media. The intensity of the evanescent wave decays exponentially with distance from the surface, selectively illuminating cell features located within a few hundred nanometres of the surface. This approach enables measurement of the distance between the interface and the components of the sperm cell within the near-field. A Cartesian coordinate system with its origin fixed at the bottom left corner of the field of view (FOV) was used for tracking sperm in both 2D and 3D (Fig. 1b).

**Figure 2:**
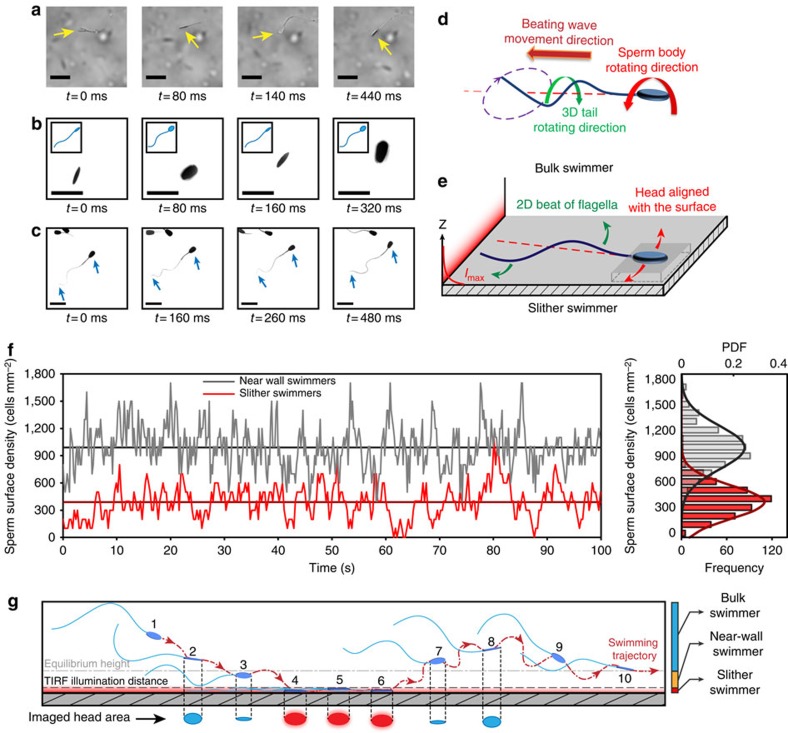
3D bulk swimming versus 2D slither swimming. Image sequences of bull sperm swimming in bulk fluid observed through (**a**) bright-field and (**b**) fluorescence microscopy, where both show continuous sperm head rotation. (**c**) Sequence of TIRF microscopy images showing a non-rotating, surface-aligned motion of the sperm head confined within a few hundred nanometres of the glass surface. The sperm tail also appears as a continuous line within the 1-μm depth of field. (**d**) Schematic of sperm swimming in bulk liquid, where the torque resulting from the 3D helical beating pattern of sperm's flagellum is balanced by counter-rotation of the sperm head. (**e**) Schematic of sperm in the observed slither mode, where both the head and the tail are confined to a 2D plane parallel to the surface with no rotation. (**f**) Sample of sperm surface density fluctuations for both near-wall swimmers and slither swimmers, with the full histogram and probability distribution function (PDF) shown on the right. (**g**) Schematic showing a typical transition from bulk swimming to slither swimming modes, with head areas shown below as would be imaged by traditional (2–3, 7–8, blue) and TIRF (4–6, red) microscopy. Scale bars, 20 μm; the image was inverted and contrast-adjusted for clarity.

Figure 2 shows a sequence of images for bull sperm swimming both in bulk fluid and within sub-micron distances from the surface. For a bulk swimmer, rotation of the sperm body around its axis results in repeating variation of the observed head area in both the bright-field images (Fig. 2a; Supplementary Movie 1) and fluorescent images (Fig. 2b; Supplementary Movie 1). Specifically, the average head area aligned with the imaging plane was 42±10 μm^2^ (mean±s.d.), a variation of 24% (*n*=503). The repeating rotation of the sperm body observed for the bulk swimmer is due to propagation of a 3D helical wave along the flagellum (Fig. 2d)^42^. This helical wave is generated by the established mechanism of the dynein motor for a 9+2 eukaryotic axoneme structure^43^,^44^. In contrast, sperm swimming near the glass surface, as imaged with TIRF microscopy, shows a more consistently aligned head area of 45±6 μm^2^ (*P*<0.01 with *t*-test), a variation of 13% (*n*=3,277; Fig. 2c; Supplementary Movie 1). The sperm tail also appears as a continuous line within ∼1-μm TIRF depth of field, indicating a high degree of confinement for the comparatively very long structure (50–80 μm). These results reveal that the motion of the whole sperm cell is highly surface-confined in these cases, with both the head aligned and the flagellum oscillating in the 2D plane (Fig. 2e).

This slither swimming mode was found to be intermittent, lasting 1,119±86 ms (*n*=85, Supplementary Fig. 1) for bull sperm in raw semen, during which each sperm traversed 68.4±3.4 μm. Figure 2f shows representative fluctuations in sperm surface density for both slither swimmers (sperm exhibiting a 2D swimming mode within 1 μm of the surface) and all near-wall swimmers (sperm within ∼4 μm distance from the surface), together with the histogram and probability distribution function. The surface density of cells decreases from 990±230 cells mm^−2^ (*n*=4,952) for near-wall swimmers, to 390±180 cells mm^−2^ (*n*=1,950) for slither swimmers (*P*≤0.0001 with a *t*-test). The results indicate that ∼39% of sperm within 4 μm of the wall were in slither swimming mode. Tracking the sperm surface density over time indicates the sperm in this region switch in and out of slither swimming mode at a rate of 480 cells s^−1^ mm^−2^ (*n*=498).

A schematic showing a typical transition from bulk swimming to slither swimming modes is shown in Fig. 2g with the head areas imaged by traditional (2–3, 7–8, blue) and TIRF (4–6, red) microscopy. Near the surface, the flow field induced by sperm is asymmetric with the no-slip wall boundary dampening the fluid velocity^24^,^28^. The asymmetry in the flow field results in a net force on the sperm towards the surface. Once the head is aligned with the surface, several factors could contribute to maintaining this orientation: (i) resistance to rotation of the head due to presence of the surface^32^, (ii) higher drag force acting on the sperm head area from the wall side^45^ and (iii) confinement of the entire tail-beating pattern within the 2D plane, as found here. Departure from the surface could be achieved by tilting the plane of the flagellar wave relative to that of the head^32^ or inducing lift due to the shape and orientation of the sperm head^30^. These observations suggest a distinct, intermittent 2D slither swimming mode exhibited by sperm near boundaries.

**Figure 3:**
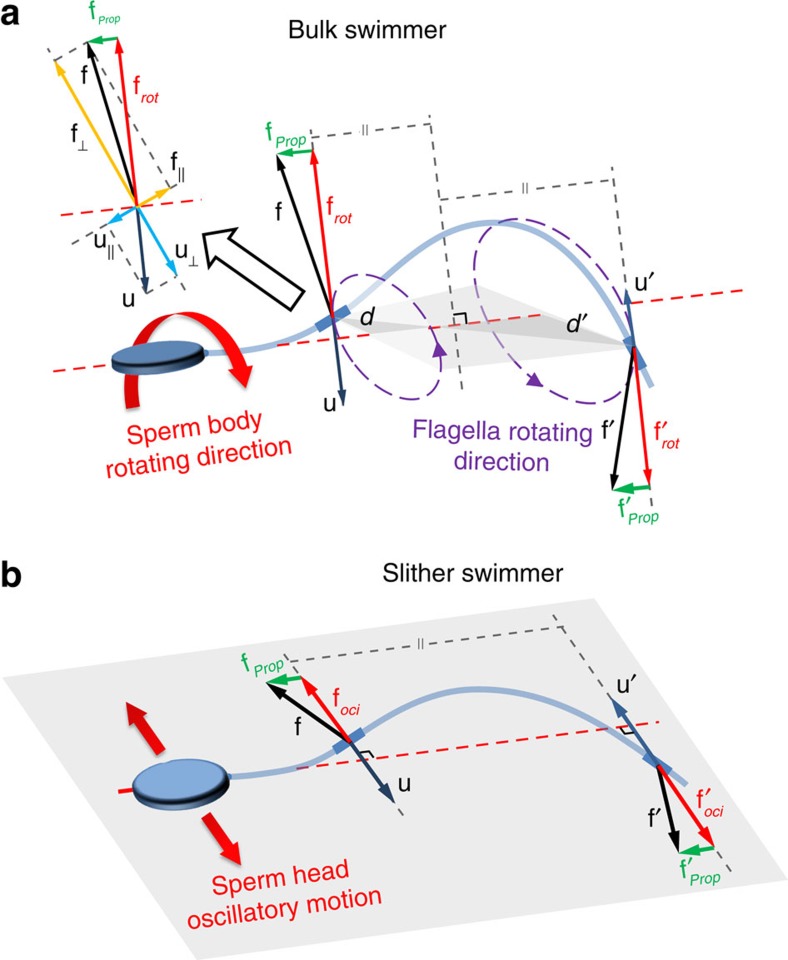
Schematic of drag-based sperm locomotion in bulk swimming and slither swimming modes. (**a**) Bulk swimmer sperm propulsion. The net drag force, **f**, acting on each segment of flagellar helix has a propulsive, **f**_prop_, and rotational component, **f**_rot_. The result is propulsion with continuous rotation in opposite direction of the flagellar wave. (**b**) Slither swimmer propulsion. The net drag force, **f**, on each segment has a surface-aligned propulsive component, **f**_prop_, and an oscillating perpendicular component, **f**_oci_, which also lies in the 2D plane of the surface. With all forces acting within the same plane, slither swimmers achieve forward progression with no rotation.

Figure 3a schematically illustrates drag-based sperm propulsion for bulk swimmers. The ratio of normal to tangent drag (**f**_⊥_/**f**_||_) acting on each segment of flagellum is higher than the same ratio of normal to tangent velocity (**u**_⊥_/**u**_||_)—a drag anisotropy that enables a forward propulsive force, **f**_prop_, in slender bodies^13^,^46^. The net drag force, **f**, acting on each segment of the flagellar helix also has a rotational component, **f**_rot_, that continuously counter-rotates the cell^46^ (Fig. 3a). Our experiments indicate that the locomotion mechanism for a slither swimmer is distinct, as schematically illustrated in Fig. 3b. The flagellar oscillation is 2D, confined within the plane of the surface. Surface proximity increases the tangent and normal drag coefficient^47^, resulting in an increase in both propulsive forces and friction. Higher lateral drag forces also dampen the transverse component of the flagellar wave and may play a role in restricting the 3D wave into a planar wave. The net drag force is made up of a propulsive component, **f**_prop_, and an oscillating perpendicular component, **f**_oci_—both of which lie in the 2D plane of the surface. Thus, the absence of out-of-plane forces enable surface-aligned motion of the sperm, without rotation.

### Preferential circling direction in slither swimming mode

**Figure 4:**
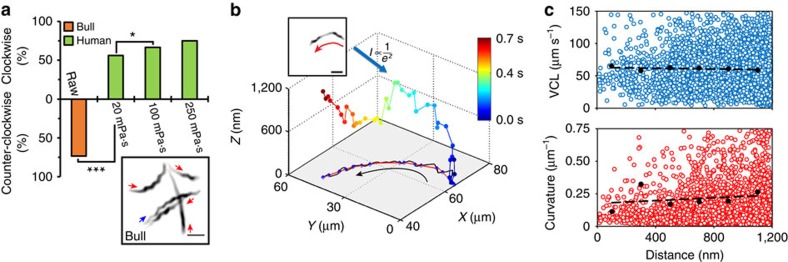
TIRF characterization of the slither swimming mode. (**a**) Preferential circling direction of bull and human sperm in slither swimming mode (*n*≥126). An overlay of consecutives images taken at 50 frames per second showing swimming trajectories of five bull sperm shown in the inset. Red and blue arrows indicate counter-clockwise and clockwise trajectories, respectively. (**b**) A reconstructed trajectory of a bull sperm and its projected trajectory in the 2D plane of the surface (out-of-plane axis is magnified × 40). The colour corresponds to time, as shown in the legend, and the black and red lines show the projected swimming trajectory and average path, respectively. The corresponding overlay of TIRF microscopy images is shown in the inset. (**c**) Curvilinear velocity (top) and local curvature (bottom) measured as a function of distance from the surface (bull sperm). Open circles represent local data points and the solid black circles are averages binned over 200-nm intervals. *P* values were determined by a one-tailed *z*-test, *P≤0.05 and ***P≤0.001. Scale bars, 20 μm.

Circling direction of bull and human sperm in slither swimming mode is shown in Fig. 4a. Consecutive TIRF microscopy frames for each individual sperm were overlaid to reconstruct swimming trajectories, as shown in the inset in Fig. 4a (also see Supplementary Fig. 2a). In more than 74% of cases (*n*=170), bull sperm in slither swimming mode follow counter-clockwise trajectories in raw semen when observed from below. In contrast, a clockwise preference was observed for human sperm. Specifically, slither swimming human sperm follow clockwise trajectories in more than 56% of cases (*n*=128, *P*≤0.001 with a *z*-test) when swimming in media with a viscosity of 20 mPa·s (all viscosities are indicated as nominal values at 20 °C according to the manufacturer's specification, measured values at 37 °C are provided in Methods). The preference of human sperm to follow clockwise trajectories increased with viscosity to 68% (*n*=126, *P*≤0.05 with a *z*-test) and 75% (*n*=126, *P*=0.072 with a *z*-test, not statistically significant) in media with viscosities of 100 and 250 mPa·s, respectively. This preferential circling direction for a slither swimmer is attributed to the inherent tendency of the flagellar wave in bull and human sperm to rotate in counter-clockwise and clockwise directions, respectively (as viewed from behind). These rotational senses result from the relative order of sliding of microtubule doublets within the flagellum of each species. Although the structure is similar in both human and bull sperm, the sequence of activation/deactivation of dynein arms, or handedness^30^,^48^, ultimately determines the rotational direction^49^. Handedness varies between species and to a lesser extent between cells^32^,^50^,^51^. In addition, the bend of planar wave is larger in one direction^30^,^39^, and the level of this bend asymmetry contributes to the curvature of the swimming path and circling direction observed for slither swimmers.

### Kinematics of sperm motion in slither swimming mode

In addition to imaging sperm motion in the 2D plane, TIRF imaging was applied to track the sub-micron distance between the surface and the sperm head for slither-mode swimmers. Specifically, the average intensity over the sperm head area provided a measure of distance from the surface. Figure 4b shows a typical trajectory (multicolour line) including both in-plane and out-of-plane components, with the out-of-plane axis amplified 40 × . While the overall motion is highly planar, we observe variations in the gap between the sperm head and the surface, within 1 μm of the surface (Supplementary Fig. 2b). For this representative case, the sperm moves ∼500 nm away from the surface over ∼0.5 s, and returns. Two aspects of this trajectory are noteworthy: (i) the timescale corresponds to several (∼5–10) flagellar beats; and (ii) the velocity in the out-of-plane dimension is quite slow, ∼7%, of that in the plane (Supplementary Fig. 2c). Thus slither-mode swimmers exhibit highly confined planar motion, with intermittent surface-normal drift in the region within 1 μm of the surface.

Figure 4c shows in-plane kinematic characteristics as a function of distance from the surface. As shown, the measured curvilinear velocity (VCL) of slither swimmers was largely independent of surface proximity (Fig. 4c top; *R*^2^=0.19, *P*=0.3283 with Pearson correlation). The curvature of the sperm paths showed a slight increase with distance from the surface (Fig. 4c bottom; *R*^2^=0.54, *P*=0.0599 with Pearson correlation). This trend is in contrast to that observed previously at much larger distances (>10 μm), where the flagellar wave exhibits an inherently 3D beat pattern^25^. In the highly confined 2D slither swimming mode observed here, proximity to the wall increasingly dampens any tail rotation, resulting in equivalent, or somewhat less, curvature at very small distances.

Table 1 summarizes the key motility parameters for bull sperm swimming both in slither swimming mode and in the bulk. Slither swimming values of VCL, average path velocity (VAP), and straight line velocity (VSL) were lower by 58, 41 and 42%, respectively, as compared with bulk swimmers (*n*≥136, *P*≤0.001 with a *t*-test). These results indicate bull sperm swimming in slither mode are on the order of 50% slower than sperm swimming in bulk media under otherwise similar conditions. Linearity (LIN), wobble (WOB) and mean curvature (MCR) of the instantaneous sperm trajectory increased by 38% (*P*≤0.001 with a *t*-test), 40% (*P*≤0.001 with a *t*-test) and 10% (*P*=0.0579 with a *t*-test, not statistically significant) for slither swimmers. The amplitude of lateral head displacement (ALH) decreased significantly, from 5.61 μm for a bulk swimmer to 1.76 μm for the slither swimmer (*P*≤0.001 with a *t*-test). Beat cross frequency (BCF) was almost constant for both bulk and slither swimmers, indicating that the beating frequency of the flagellar wave, originating from the inherent beating frequency of microtubule doublets, remains similar in both modes. Taken together, these results indicate that in slither mode, bull sperm swim slower, along a more straight path, with less oscillation of the head—all of which point to swimming behaviour that is heavily damped by the increased drag force experienced near the surface.

Experiments with human sperm swimming in media with viscosity ranging from 2 mPa·s (raw semen) to 250 mPa·s showed that the tendency of human sperm to exhibit slither swimming increases with viscosity (Supplementary Movie 2). Specifically, no slither swimmers were detected in raw human semen samples over a relatively long observation time of 10 min. In contrast, slither swimming was observed for viscosities above 20 mPa·s with duration of slither swimming increasing with viscosity (viscosities in the fallopian tube can exceed 200 mPa·s (refs 6, 52)). Human sperm exhibited slither swimming mode for 872±82 ms at 20 mPa·s, this time increased to 995±95 ms and 1,201±115 ms at 100 mPa·s and 250 mPa·s, respectively (*n*=88, *P*<0.001 with a *t*-test between all three cases). In contrast, bull sperm exhibited a slither swimming mode even in raw semen with a viscosity of 2 mPa·s. The difference between bull and human sperm behaviour is attributed here to the stronger transverse component of the flagellar wave in human sperm^16^. Increased media viscosity dampens the strong transverse component, enabling human sperm to more closely approach, and align with, the surface.

**Figure 5:**
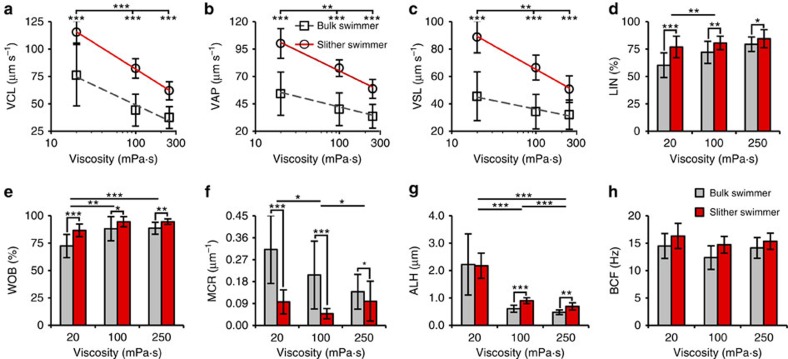
Human sperm motility parameters for slither swimming mode as compared with bulk swimming mode, as a function of viscosity. (**a**) Curvilinear velocity (VCL), (**b**) average path velocity (VAP), (**c**) straight line velocity (VSL), (**d**) linearity (LIN), (**e**) wobble (WOB), (**f**) mean curvature (MCR), (**g**) amplitude of lateral head displacement (ALH) and (**h**) beat cross frequency (BCF) for slither swimmer human sperm compared with bulk swimmer sperm in media with viscosity ranging from 20 to 250 mPa·s (*n*≥126). Human sperm swimming in slither mode showed significantly higher velocities compared with sperm swimming in bulk fluid in media with the same viscosity. Increase in viscosity negatively affect sperm motility. Values are reported as mean±s.d., and *P* values were determined by the two-tailed *t*-test, *P≤0.05, **P≤0.01 and ***P≤0.001.

Figure 5 details the motility parameters of human sperm in both slither and bulk swimming modes with media viscosities ranging from 20 to 250 mPa·s (Supplementary Movie 2). Increasing viscosity reduced the swimming velocities of both bulk and slither swimmers. However, as compared with bulk-swimming human sperm under similar conditions, slither swimmers exhibited VCL, VAP and VSL increases between 52–86%, 74–93% and 58–95% (min—max), respectively (Fig. 5a–c, *n*≥126, *P*≤0.001 with a *t*-test). Similar to bull sperm, LIN and WOB for human sperm were higher for slither swimming as compared with bulk swimming (Fig. 5d,e), indicating that sperm follow straighter trajectories in slither mode. Increasing viscosity also increased both LIN and WOB, although the differences between slither and bulk swimmers become less significant at higher viscosities. The MCR of the instantaneous sperm trajectory, characterized by MCR, decreased considerably for slither swimmers as compared with bulk swimmers (Fig. 5f), indicating an increase in symmetry of the flagellar wave^39^. This significant reduction in oscillations may contribute to the increased velocity and LIN seen in slither mode. With respect to the amplitude of head displacement, our results show a strong negative correlation between ALH and viscosity for both swimming modes, and only slightly higher ALH values for slither swimmers at higher viscosities (Fig. 5g). Regarding BCF, it was relatively insensitive to viscosity, with similar values obtained for both swimming modes (Fig. 5h)—as observed for bull sperm. Taken together, these results show that in slither mode, human sperm swim markedly faster and straighter. These results are supported by previous numerical studies^44^,^53^ showing that, for single flagellated microswimmers such as sperm, planar waves result in higher swimming velocities as opposed to helical waves (when amplitudes are unchanged).

### Synchronized motion in slither swimming mode

**Figure 6:**
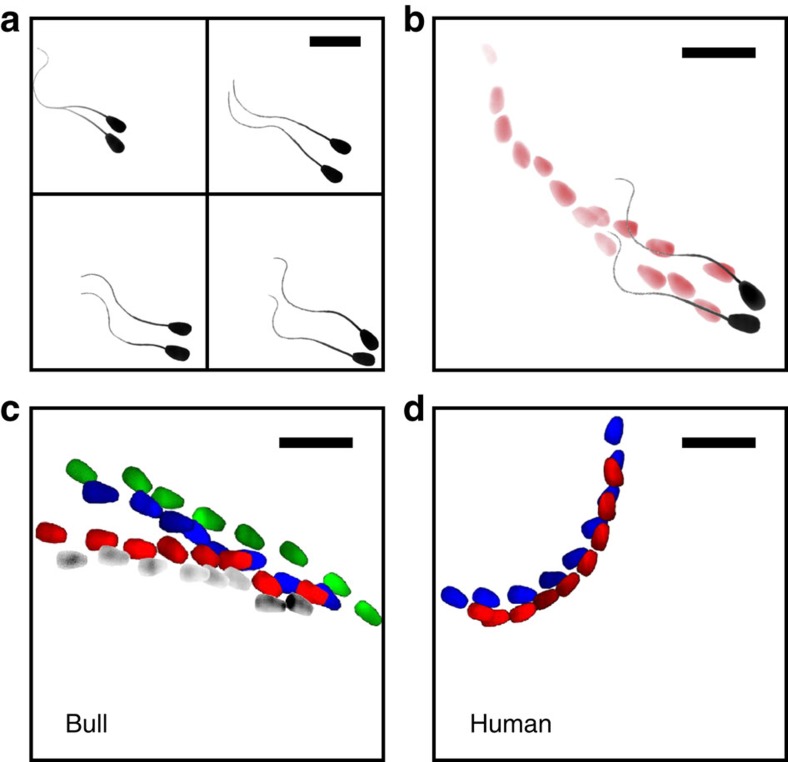
Synchronized motion of sperm in slither swimming mode. (**a**) An image sequence showing a pair of synchronized slither swimming bull sperm, adopting a shared beating frequency and flagellar waveform and (**b**) their overlaid trajectories. Synchronized motion of (**c**) four bull sperm and (**d**) two human sperm in the slither swimming mode. The head of each individual sperm is colour coded along its swimming path. Scale bars, 20 μm. The image was inverted and contrast-adjusted for clarity.

Synchronized motion of sperm has been observed for a variety of species including bull^38^, sea urchin^33^ and human^54^. Previously, synchronization has been found to result from hydrodynamic coupling of proximate flagella, with increasing synchronization in the near-wall region^33^,^38^,^55^. Flagellar synchronization was observed here to occur for both bull and human sperm in slither swimming mode. Figure 6a is an image sequence of a pair of synchronized bull sperm in a slither swimming mode with a shared flagellar beating frequency and waveform. The synchronized sperm motion was observed to occur for both initially conjoined sperm (Fig. 6b) and for sperm swimming up to 15 μm away from each other and for up to four individual sperm at a time (Fig. 6c,d; see Supplementary Movies 3 and 4). Over 40% of bull sperm (212 of 526 observed) showed synchronized motion in slither mode, while less than 1% showed synchronization in the bulk (4 of 1,100 observed). We attribute increased synchronized pairing in slither mode to the alignment of the sperm heads, flagellar waves and swimming trajectories in the surface plane, in contrast to unconstrained 3D motion in the bulk. These results indicate that spatial confinement inherent to slither swimming more readily facilitates synchronization.

## Discussion

We used TIRF microscopy to study the hydrodynamics of bull and human sperm locomotion within one micron from the surface. Our results reveal a distinct 2D locomotion mode—slither mode—for sperm near the surface, whereby the sperm head aligns with the surface and the flagellum oscillates in the 2D plane. This swimming mode is distinct from established 3D bulk swimming modes for which the flagellum propagates a helical wave and the sperm head continuously rotates. In slither mode, both high drag force and steric repulsion from the surface suppress the inherent 3D beating pattern of the flagellum. The absence of out-of-plane forces for the slither swimmer enable surface-aligned motion of the sperm, without rotation. This slither swimming mode was found to be intermittent, lasting ∼1 s for bull sperm in raw semen, during which each sperm traversed an average of 68.4 μm. Of all sperm within 4 μm of the wall, 39% were in slither swimming mode, and switched in and out of slither swimming mode at an average rate of 480 cells s^−1^ mm^−2^.

The planar waveform observed here is in contrast to common mechanism of the dynein motor activity for a 9+2 axoneme structure^4^,^43^. It has been suggested that microtubule doublets operate in two groups, activated alternately at appropriate set points^30^,^56^,^57^ to generate a planar bend. In general, the mechanisms that trigger the switch between a helical and planar waveform are not well understood^1^,^48^. Results here indicate that the combination of shear force and wall proximity can trigger a fully planar wave. Notably, drag force is increased both by the bulk viscosity of the swimming media^52^,^58^ and the proximity of the swimmer to the solid boundary^47^. The resulting higher force production rates of dynein arms may, in turn, act as a molecular level mechanism to regulate the dynein motor—a form of hydrodynamic regulation in the absence of any chemical stimulus.

In terms of viscosity dependence, bull sperm strongly exhibit slither swimming mode even in low-viscosity media; however, for human sperm, slither swimming prevails only at higher viscosities (≥20 mPa·s). In human sperm, strong transverse components of the flagellar wave at low viscosities (<20 mPa·s) inhibit slither swimming mode. However, an increase in viscosity dampens this transverse component and encourages slither swimming. This correlation suggests the potential utility of slither swimming for human sperm in regions of the fallopian tube where the viscosity of mucus reaches over 200 mPa·s.

With respect to speed, bull sperm are 50% slower in slither mode while human sperm are 50% faster, as compared with bulk swimmers under otherwise similar conditions. The ultimate swimming velocity in the planar slither mode is expected to depend primarily on the flagellar wave amplitude and drag forces due to the proximity of the surface. Bull sperm exhibit small chiral components of the flagellar wave in bulk swimming, and transitioning to slither mode negatively affects the swimming velocity by further decreasing the amplitude of flagellar wave oscillation, as suggested by lower ALH values. In contrast, human sperm exhibit characteristically large chiral components of the flagellar wave in bulk swimming. In human sperm, slither swimming confines the beat pattern, restricts the yaw in the trajectory and in some cases increases the amplitude of oscillations—resulting in a net increase in human sperm velocity.

For both bull and human sperm, sperm follow straighter trajectories in slither mode, as indicated by higher LIN and WOB values as compared with bulk swimming. Furthermore, slither swimmers show a preference to follow clockwise (human sperm) or counter-clockwise (bull sperm) trajectories, dictated by the rotational direction of the flagellar wave and flagellar bend asymmetry. Despite the similar 9+2 structure of axoneme in bull and human sperm, species-specific handedness in activation and deactivation of the dynein arms dictates the rotational direction of the flagellar wave, which in turn determines the trajectory curvature in slither swimming mode. Both bull and human sperm also exhibit synchronized motion in slither mode with shared flagellar waveform and frequency. Over 40% of bull sperm in slither mode showed synchronization, as compared with less than 1% in the bulk, indicating that planar confinement more readily facilitates synchronized locomotion of sperm.

In the context of reproduction, our findings suggest a surface-based sperm migration strategy in the fallopian tube that is species specific. In humans, the oviduct contains sections of relatively narrow lumen with compressed labyrinthine structures^8^,^9^, such as the isthmus and ampulla, where viscosities exceed 200 mPa·s (refs 6, 52). Slither swimming may allow human sperm to navigate faster through these highly confined and viscous regions. However, the swimming velocity in the slither mode is also highly amplitude dependent. For species with weak transverse component of the flagellar wave, such as bull, slither swimming is slower than bulk swimming. The larger anatomy of the bovine fallopian tube^8^,^9^ may favour faster bulk swimming, perhaps at the expense of slither swimming speed. Taken together, these findings show that sperm exhibit a distinct 2D slither swimming mode when within a micrometre of the surface. For humans, the implication is that slither swimming is a distinct swimming mode, which is suited to the highly viscous and confined regions of the fallopian tube.

## Methods

### Sperm sample preparation

Cryogenically frozen human semen was purchased in 1-ml vials from ReproMed Ltd (Toronto, Canada) and stored in liquid nitrogen. All donors provided consent for research participation in accordance with regulations of the Assisted Human Reproduction Act. Human semen vial was thawed for 5 min in a 37-°C water bath before the experiment. Human semen with 50 million sperm per millilitre concentration and 40% motility was used in the experiment. Bull semen was purchased in 500 μl straws from ABS Global Inc. (Ontario, Canada) and stored in liquid nitrogen. Before the experiment, bull semen was thawed in a water bath at 37 °C and removed from the straw using an artificial insemination syringe. The bull sperm concentration in the semen sample was measured to be ∼40 million sperm per millilitre with 50% vitality and 50% motility. HEPES-buffered salt solution (135 mM NaCl, 5 mM KCl, 12 mM D-glucose, 25 mM HEPES, 0.75 mM Na_2_HPO_4_·2H_2_O) supplemented with 1 mg ml^−1^ poly(vinyl alcohol) with 0.5, 0.875 and 1.125% methyl cellulose (MC) (M0512; Sigma-Aldrich Corp., MO) was used to examine sperm motion in different fluid rheologies with nominal viscosities of 20, 100 and 250 mPa·s, respectively, at 20 °C. A Brookfield LVDV-E digital viscometer (Brookfield Engineering Laboratories, Inc., MA, USA) with spindle LV2 at 100 r.p.m. was used to measure the viscosity of the MC media at 37 °C. The respective viscosity of buffers with 0.5, 0.875 and 1.125% MC were measured to be 18.95±0.15, 88.53±1.40 and 210.50±3.72 mPa·s at 37 °C. The viscosity values used in differentiating the buffers in the text are nominal values at 20 °C as in the manufacturer's guideline. Live sperm was fluorescently labelled by the addition of 50 μl of 50-fold-diluted SYBR 14 dye (Comp A, LIVE/DEAD sperm viability kit, L-7011; Invitrogen, NY, USA) to 500 μl of semen followed by incubation for 10 min at 37 °C. The experiments were conducted within 10 min of sperm staining and the sample was kept at 37 °C for all time. Before each experiment, 100 μl of stained semen was transferred to a glass-bottom WillCo dish (GWSt-3522, WillCo Wells, The Netherlands) for imaging purposes.

### Microscopy

An inverted fluorescence microscope (DMI 6000B, Leica) with a charge-coupled device camera was used to capture × 40 magnification (numerical aperture (NA)=0.60, HCX PL Fluotar) image sequences in bright field and green fluorescence. A PCO 1200 high-speed camera (PCO AG, Kelheim, Germany), mounted on the inverted microscope, was used to capture image sequences with 50-Hz frame rates. The focal plane was adjusted to be away from the surfaces to ensure that bulk swimmer sperm were imaged. Each imaging experiment was performed for 3 min.

### TIRF microscopy

An objective-based TIRF microscopy set-up was used to image swimming trajectories of bull sperm near the glass-bottom surface of a WillCo dish. TIRF microscopy was performed on a home-built TIRF microscopy system integrated with an Olympus FluoView 500 confocal microscope using a nIX-70 base (Olympus, Canada) using a high NA × 60 oil-immersion objective (NA=1.45, Olympus). A thin layer of index-matching oil with *n*_oil_=1.515 was used to optically couple the objective to the glass surface of the WillCo dish. Excitation was achieved using an analogue-modulated 473-nm diode laser (DHOM-L-150 mW, Suzhou Daheng Optics & Fine Mechanics Co., Ltd, China). Fluorescent images are captured at a 50-Hz frame rate using a eXcelon equipped Evolve 512 EMCCD camera (Photometrics, AZ, USA) in a water-cooled mode using Micro-Manager (version 1.4.19). Imaging was performed over a 3-min period.

TIRF microscopy benefits from excellent signal-to-noise ratio with illumination limited to a sub-diffraction-limited volume in the near-field region, which makes it ideal to study live cell dynamics at surfaces^40^. In TIRF microscopy, the total internal reflection of the incident beam from the glass–liquid interface results in an electromagnetic wave, named an evanescent wave, to propagate horizontally in the liquid phase along the interface. The intensity of the evanescent wave, *I*_*z*_, decreases exponentially with distance from the surface as:


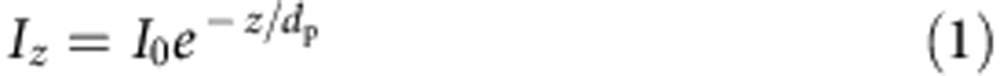


where *z* is the perpendicular distance to the interface, *I*_0_ is the intensity at *z*=0 (maximum intensity) and *d*_p_ is the penetration depth^59^,^60^. The penetration depth defined as:


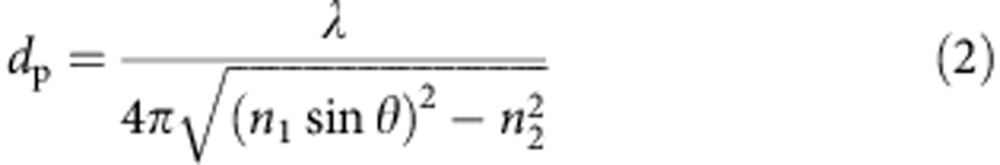


where *λ* is the wavelength of the incident light and *θ* is the incident angel. By neglecting the near-field effect on collected fluorescence intensity, the perpendicular distance to the interface for a known intensity can be calculated as *z*=−*d*_p_ ln(*I*_*z*_/*I*_0_).

In our experiments, the refractive indices of bull semen and glass surface were *n*_2_=1.3635 and *n*_1_=1.5255, respectively, resulting in a critical angle, *θ*_c_=63.35. The refractive index of bull semen was measured using a Standard Abbe Refractometer (Edmund Optics Inc., NJ, USA). For an excitation wavelength of 473 nm, the penetration depth is ∼75 nm at the maximum incident angle of 71.9°, using an NA of 1.45 (Supplementary Fig. 3). The incident angle of *θ*=63.5° was used in the experiments, resulting in *d*_p_=549 nm. In this study, the TIRF configuration enabled imaging of sperm located within 1,200 nm of the surface.

### Image analysis

Image processing was performed to extract quantitative information related to sperm swimming characteristics in bulk liquid and near the glass surface. All fluorescence and TIRF microscopy images were background corrected. Sperm head area was tracked manually in the time-sequence images using the freely available image processing software ImageJ. Centroids of the sperm heads were used to calculate the sperm position in the XY plane. For TIRF microscopy images, the average intensity over the sperm head area was used in a custom-written script in Matlab to calculate the distance from the glass surface (Supplementary Fig. 3b), assuming that the closest distance that sperm can swim is 10 nm. To ensure the uniform distribution of fluorescent dye for different cells, the average intensity over the head area was measured for different sperm (*n*=274) in a sequence of fluorescence images, indicating less than 5.7% fluctuation around a fixed value. Only sperm that both enter from one side of the FOV and exit from the other side, or sperm that both appears and disappears in the FOV within a minimum of 15 captured images were considered in the image analysis.

A custom-written script in Matlab was used to analyse the motility parameters and reconstruct the swimming trajectories of sperm in both 3D and 2D. Motility parameters were calculated to be compatible with the current standards used by computer-aided sperm analysis systems^61^,^62^. The motility parameters were defined as: (i) Curvilinear velocity (VCL): the sum of incremental distance between each two consecutive sperm position in the trajectory/the associated time difference; (ii) average path velocity (VAP): time-average velocity of the sperm along its average path; (iii) straight line velocity (VSL): the distance between the first and the last sperm tracking point in each trajectory/total duration of the track segment; (iv) LIN: VSL/VCL; (*v*) WOB: VAP/VCL; (vi) MCR: time-average curvature of sperm along its actual trajectory; (vii) ALH: time-average deviation of the sperm head from the average path; and (viii) BCF is the frequency at which the 2D projection of sperm trajectory cross the average path trajectory. These values were averaged over each trajectory, and their mean over number of recorded trajectories in each case was calculated as the final value for each parameter (*n*>1,400 data points). To select the appropriate statistical test, we verified that the data follow a normal distribution (Supplementary Fig. 4). A statistical *t*-test was used to analyse numerical variables with normal distribution. A *z*-test was used to analyse statistical significance for categorical variables (circling direction), when applicable. *P*<0.05 was considered as significant.

### Code availability

Matlab version 8.4.0.150421 (R2014b) was used to generate custom scripts to analyse the motility parameters and reconstruct the swimming trajectories of sperm in both 3D and 2D. Please contact David Sinton, at sinton@mie.utoronto.ca, for access.

## Additional information

**How to cite this article:** Nosrati, R. *et al.* Two-dimensional slither swimming of sperm within a micrometre of a surface. *Nat. Commun.* 6:8703 doi: 10.1038/ncomms9703 (2015).

## Supplementary Material

Supplementary Information Supplementary Figures 1-4

Supplementary Movie 1 Compilation comparing bulk swimming to slither swimming bull sperm. (a) Bulk swimming sperm imaged with bright-field microscopy; (b) bulk swimming sperm imaged with fluorescence microscopy; and (c) Slither swimming sperm imaged using TIRF microscopy. All videos are in real-time, in raw semen with a viscosity of 2 mPa・s. Scale bars are 20 μm and the images were inverted and contrast-adjusted for clarity.

Supplementary Movie 2 Compilation comparing slither swimming human sperm at different viscosities. (a) 20 mPa・s; (b) 100 mPa・s; and (c) 250 mPa・s. All videos are in real-time, as imaged with TIRF microscopy. Scale bars are 20 μm and the images were inverted and contrast-adjusted for clarity.

Supplementary Movie 3 Synchronized motion of bull sperm in slither mode. Videos are in real-time, as imaged with TIRF microscopy. Scale bars are 20 μm and the images were inverted and contrast-adjusted for clarity.

Supplementary Movie 4 Synchronized motion of human sperm in slither mode. Videos are in real-time, as imaged with TIRF microscopy. Scale bars are 20 μm and the images were inverted and contrast-adjusted for clarity.

## Figures and Tables

**Table 1:** Swimming characteristics of bulk swimming versus slither swimming bull sperm (2 mPa·s).

**Parameters**	**Bulk swimmer (*****n*****=136)**	**Slither swimmer (*****n*****=170)**
VCL (μm s^−1^)***	176.0±13.2	74.7±16.4
VAP (μm s^−1^)***	100.6±7.8	59.0±11.0
VSL (μm s^−1^)***	94.4±8.5	54.7±11.0
LIN (%)***	54±4	74±8
WOB (%)***	57±3	80±8
MCR (μm^−1^)	0.19±0.06	0.21±0.09
ALH (μm)***	5.69±0.40	1.71±0.81
BCF (Hz)	18.8±1.7	17.5±2.3

ALH, amplitude of lateral head displacement; BCF, beat cross frequency; LIN, linearity; MCR, mean curvature; WOB, wobble.

Motility parameters were calculated as curvilinear velocity (VCL), average path velocity (VAP), straight line velocity (VSL), LIN, WOB, MCR, ALH and BCF. Values are reported as mean±s.d., and *P* values were determined by the two-tailed *t*-test between bulk swimmers and slither swimmers, ***P≤0.001.
