# Supplementary material for: Two-dimensional slither swimming of sperm within a micrometre of a surface
Source: Nat Commun. 2015 Nov 10;6:8703. doi: 10.1038/ncomms9703 (PMC4667638; doi:10.1038/ncomms9703)
Supplement: Supplementary Information — Supplementary Figures 1-4 [file ncomms9703-s1.pdf]

Supplementary Figures

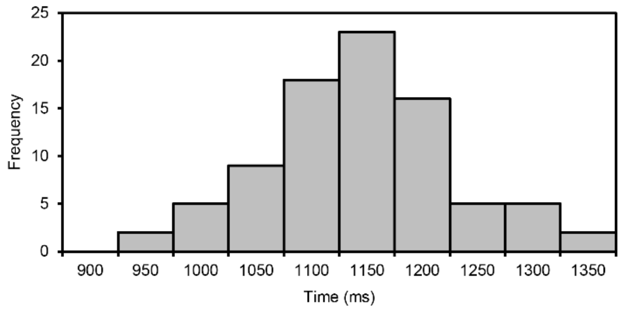

Supplementary Figure 1 | Frequency histogram of time that bull sperm exhibited slither swimming mode. Bull sperm exhibited slither swimming mode for 1119±86 ms (n = 85).

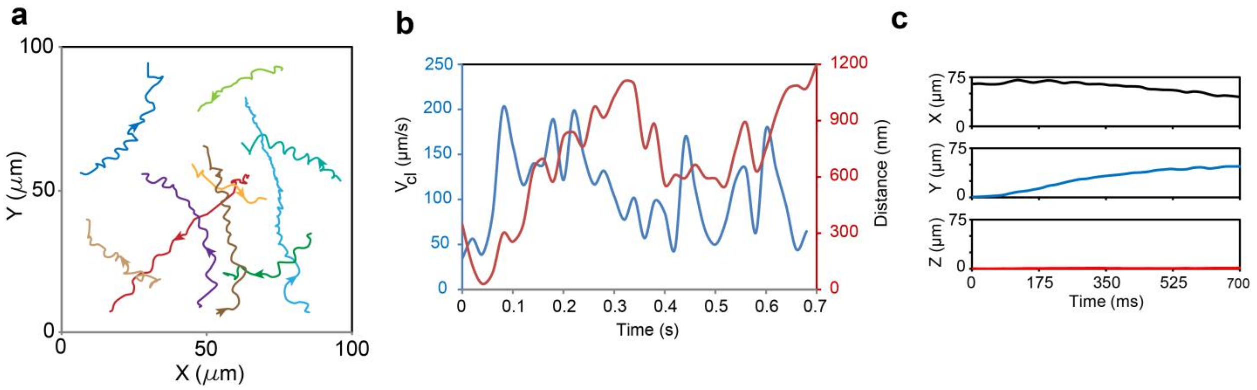

Supplementary Figure 2 | Calculated trajectories and velocities of typical slither swimming bull sperm. (**a**) A typical indication of tracked swimming trajectories of ten sperm projected in the 2D plane. (**b**) Instantaneous curvilinear velocity and distance of the tracked sperm versus time for a typical trajectory shown in Fig. 4b. (**c**) Sperm movement in X, Y, and Z direction over time indicating the relatively minor movement of sperm in the Z direction, normal to the surface, compared to other two directions.

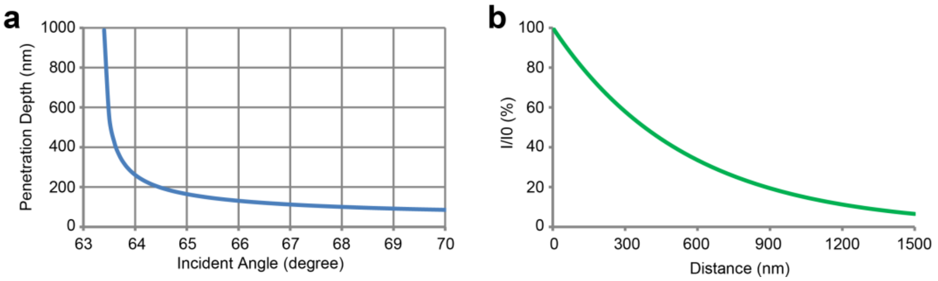

Supplementary Figure 3 | Theoretical characteristics of the TIRF microscopy setup. (**a**) The penetration depth of the TIRF microscopy setup as a function of incident angle. (**b**) Normalized intensity of the evanescent wave as a function of distance from the surface for the incident angle of 63.5°. I_0 is the maximum intensity at the interface.

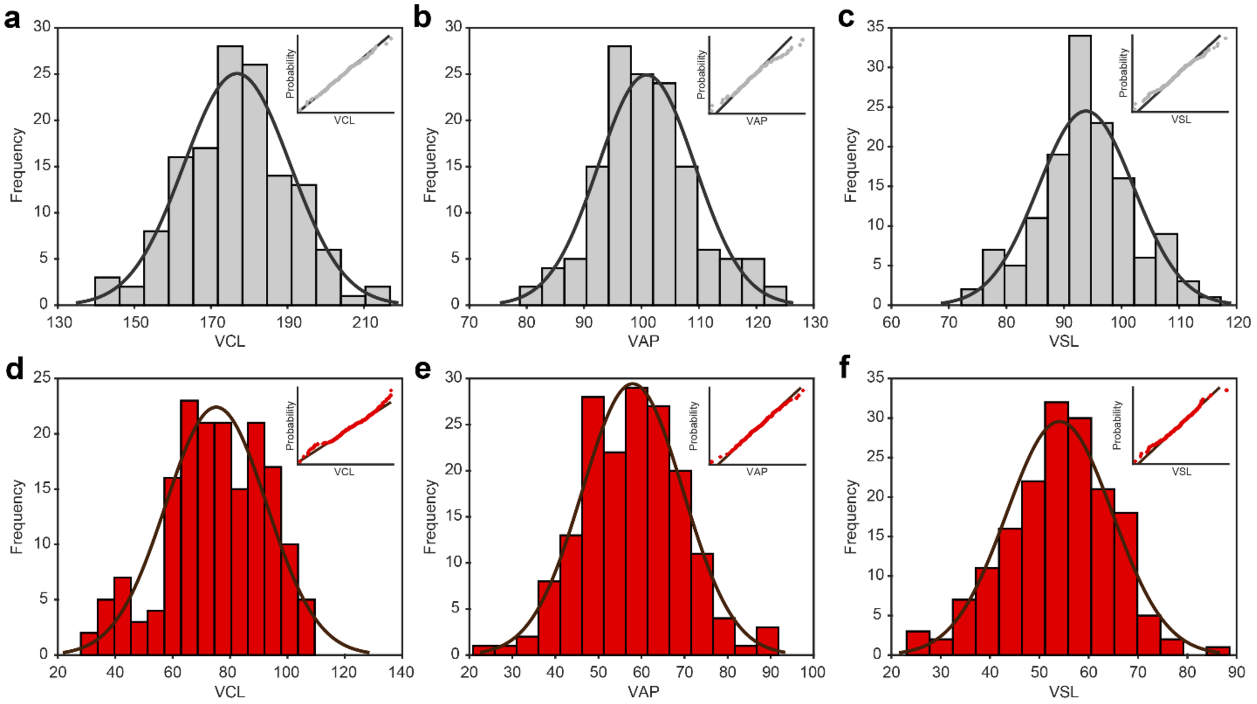

Supplementary Figure 4 | Verification of normal distribution of bull sperm velocities. Frequency histogram of (**a**) curvilinear velocity (VCL), (**b**) average path velocity (VAP), and (**c**) straight line velocity (VSL) for bulk swimming sperm. Frequency histogram of (**d**) VCL, (**e**) VAP, and (**f**) VSL for slither swimming sperm. The continuous line indicates a perfect normal distribution. Normal probability plots shown inset with data points indicated with +.
